# Editorial: Innovative paradigms of accompaniment in healthcare: humanizing practices

**DOI:** 10.3389/fpsyg.2026.1966100

**Published:** 2026-09-24

**Authors:** Paula Crespí

**Affiliations:** Accompaniment Institute, Universidad Francisco de Vitoria, Madrid, Spain

**Keywords:** accompaniment, education of healthcare professionals, family, healthcare, human-centered, humanizing practices, patients, students

## Introduction

In recent decades, accompaniment has attracted growing interest in healthcare settings within a context marked by the search for greater humanization of care (Henao-Castaño et al., 2021; Reyes-Téllez et al., 2024) and the need to promote the integral development of both those being accompanied and those who accompany others (Crespí and López, 2023). In healthcare contexts, various relational approaches have been developed to support patient care, education, and professional support. These include the deliberative clinical relationship (Seoane, 2016), mentoring and tutoring (Álvarez-Montero et al., 2023), coaching (Todorova, 2022), and counseling (Hack et al., 2023). Although all of these approaches provide valuable contributions, they are generally oriented toward specific objectives, such as decision-making, problem-solving, performance improvement, or competence development. By contrast, accompaniment is understood as an intentional pedagogical action that helps individuals gain deeper self-knowledge and make decisions that foster their personal development (Crespí and López, 2023). From a broader perspective, it constitutes a relational process aimed at the integral development of the person and grounded in respect for human dignity and freedom (González-Iglesias and de la Calle Maldonado, 2020; Viñado Oteo and Miró López, 2022).

Its relevance is particularly evident in light of phenomena such as burnout among healthcare professionals (Nagarajan et al., 2024), perceptions of a lack of compassion within healthcare systems (Henao-Castaño et al., 2021; Lown et al., 2011, 2017), and the ethical challenges associated with research involving human participants (Pietilä et al., 2019; Resnik, 2024).

The 14 papers included in this Research Topic demonstrate the growing international interest in accompaniment as a strategy to humanize healthcare, support patients and families, and strengthen the education and professional development of healthcare practitioners.

## Conceptualizing and defining accompaniment

A recurring concern throughout this Research Topic was to clarify what accompaniment actually means, given the broad and often inconsistent use of the term across disciplines. Antón-Rodríguez et al. conducted a systematic review and conceptual analysis based on PubMed and Embase records, proposing an operational definition of accompaniment as a personal and ethical relationship aimed at human development through a shared process that fosters a humanized social environment. They identified palliative care as a model case encompassing all the defining attributes of accompaniment.

Complementing this empirical synthesis, Núñez-Sánchez et al. offered a theoretical and conceptual reflection on accompaniment as a fundamental relational style underlying integral care, examining its assumptions, attitudes, and scope within healthcare settings. López González et al. extended this conceptual work into the educational domain through a scoping review of educational accompaniment, identifying four distinct paradigms: psychosocial, personalist, socio-educational, and competency-based. Their work proposed an integrated definition focused on fostering autonomy, maturity, and a fulfilling life.

Finally, Orón Semper and Antón Rodríguez approached the concept from its opposite pole by conducting a systematic review and conceptualization of disengagement, understood as a progressive deterioration of relationships arising from a perceived loss of meaning in commitments and interpersonal bonds. They argued that understanding this process is essential for designing early detection strategies and humanizing accompaniment interventions in clinical, educational, and organizational contexts.

## Accompaniment in palliative and chronic care

Several contributions examined how accompaniment operates in the context of serious or chronic illness. Ricci et al. proposed a conceptual framework that positions hope as a form of moral accompaniment in palliative care. Drawing on Snyder's hope theory, understood as the combination of agency and pathways thinking, they argued for its indirect therapeutic value at the psychophysical level.

Addressing a different population, Chen et al. conducted a qualitative study exploring the need for accompaniment services during medical visits among older adults with chronic diseases and their family members. Using Existence, Relatedness, and Growth (ERG) theory to structure their interview guide, they identified specific and often unmet support needs within this population.

Shifting the focus to the professionals who provide such accompaniment, Dong et al. employed latent profile analysis to identify two distinct groups among 316 palliative care nurses, one characterized by lower-quality and the other by higher-quality accompaniment. Their findings showed that nurses' attitudes toward palliative care, sense of meaning in life, and competence in coping with death were the key factors distinguishing the two profiles.

Complementing this human-centered perspective on accompaniment, Palomino-Lázaro et al. conducted a scoping review of animal-assisted therapy in palliative care. Synthesizing evidence from 16 studies, they found that structured interactions with therapy animals may reduce pain, anxiety, and depression, while also promoting social interaction and emotional comfort. Their findings suggest a complementary, non-human form of accompaniment for patients facing terminal illness.

## Family and caregiver accompaniment

A distinct group of studies focused on accompaniment directed not at patients themselves but at their family caregivers. Liu et al. evaluated a 12-week multidisciplinary accompaniment intervention for family members of ostomy patients, finding significant improvements in caregiving capacity, as well as reductions in negative emotions and social isolation, with the latter acting as a partial mediator of these effects. In a related line of research, this time centered on patients rather than caregivers, Zhou et al. examined the effects of high-quality nursing accompaniment among patients with acute myocardial infarction. Their findings showed improvements in sleep quality, reductions in anxiety and depression, enhanced quality of life, and fewer complications when compared with conventional nursing care.

## Accompaniment in populations with specific needs

Beyond general patient populations, this Research Topic also addressed accompaniment tailored to specific clinical profiles. San José Cáceres et al. conducted a qualitative study of the Comprehensive Medical Care Unit for People with Autism Spectrum Disorder (AMITEA), gathering the experiences of patients, caregivers, and healthcare professionals through focus groups structured according to the Picker model of patient-centered care.

Their findings highlighted the value of respectful, personalized, and sensory-adapted care. However, they also identified persistent gaps in coordination beyond specialized units, in the transition to adulthood, and in professional training outside these programs. These results underscore the need to extend humanized accompaniment practices more broadly across healthcare systems.

## Accompaniment in the education of healthcare professionals

Finally, a substantial group of contributions examined how accompaniment can be integrated into the education of current and future healthcare professionals. Crespí et al. evaluated an integral accompaniment program for first-year university students, including those enrolled in Medicine and Health Sciences programs, and found significant improvements in participants' self-perceived intrapersonal and interpersonal competencies following the intervention.

In a related quasi-experimental study, Gallo-Giunzioni et al. assessed an educational innovation program for Psychology students based on simulated clinical scenarios carried out under the accompaniment and supervision of an instructor. The program was designed to strengthen competencies such as teamwork, communication, and ethical awareness for future clinical practice.

Adopting a broader institutional perspective, Herruzo and Castañeda-Vozmediano presented a retrospective 14-year analysis of a Comprehensive Academic Support and Mentoring Program for underperforming medical students, evaluating its impact on reducing insufficient academic performance and supporting the recovery of students at risk of dismissal.

## Conclusion

Taken together, the contributions included in this Research Topic portray accompaniment as a coherent, albeit still evolving, relational paradigm that spans the conceptual, clinical, and educational domains of healthcare. Collectively, they converge on a common premise: humanizing care requires attention not only to technical outcomes but also to the quality of the relationships through which care, support, and learning are provided, whether between clinician and patient, professional and student, or caregiver and family member.

At the same time, this Research Topic highlights the current limitations of the field. Definitions of accompaniment remain heterogeneous, empirical validation is still uneven across contexts, and most intervention studies rely on samples drawn from a single institution or country, limiting the generalizability of their findings. Future research would benefit from cross-cultural and longitudinal designs, the use of validated measurement instruments, and a closer integration of the conceptual and empirical dimensions of this literature.

We hope that this Research Topic will stimulate further dialogue and rigorous research on accompaniment as a transformative force for the humanization of healthcare practices.
